# Longevity and gender as the risk factors of methicillin-resistant *Staphylococcus aureus* infections in southern Poland

**DOI:** 10.1186/s12877-017-0442-3

**Published:** 2017-02-10

**Authors:** Monika Pomorska-Wesołowska, Anna Różańska, Joanna Natkaniec, Barbara Gryglewska, Anna Szczypta, Mirosława Dzikowska, Agnieszka Chmielarczyk, Jadwiga Wójkowska-Mach

**Affiliations:** 1 Department of Microbiology, Analytical and Microbiological Laboratory of Ruda Slaska, KORLAB NZOZ, Ruda Slaska, Poland; 2 Chair of Microbiology, Faculty of Medicine, Jagiellonian University Medical College, 18 Czysta Street, 31-121 Krakow, Poland; 3 Department of Internal Medicine and Gerontology, Jagiellonian University Medical College, Krakow, Poland; 4 Faculty of Health and Medical Sciences, Andrzej Frycz-Modrzewski Krakow University, Krakow, Poland; 5 Department of Clinical Nursing Jagiellonian University Medical College, Krakow, Poland

**Keywords:** MRSA, Longevity, Trimethoprim/sulfamethoxazole, Antibiotic resistance, Infection

## Abstract

**Background:**

The proportion of older people among the general population has risen. *Staphylococcus aureus* (SA) constitutes a significant problem. Underlying disease and functional debility, predispose the older adult to staphylococcal carriage and infection, specially bloodstream infection and pneumonia.

**Methods:**

This is a retrospective cohort study of older patients with SA infections. We analyzed a database containing the results of laboratory cultures from patients treated in 2013 for SA infections and selected 613 hospitalized and non-hospitalized people aged ≥60 years.

**Results:**

The prevalence of Methicillin-resistant SA (MRSA) were significantly different in categories of patients: from 14.1% in young old, 19.5% in old old and 26.7 in longevity. MRSA was significantly more frequently reported in cases of pneumonia, 40.4% of SA strains (*p* < 0.0001, OR 0.3, 95%CI 0.14–0.49). The nosocomial MRSA infections were more common in ICU departments: prevalence 36.8%, than in non-ICU departments: prevalence 17.3% (OR 2.8, 95%CI 1.06–7.34, *p* = 0.014). Bloodstream infections, which accounted for 6% of all infections, were more frequent in males (*p* = 0.0231, OR 2.25, 95%CI 1.098–4.604). The greatest increase in antibiotic resistance was related to trimethoprim/sulfamethoxazole (TMP/SXT), which increased to over 80% in the older study groups. All age groups demonstrated increased MIC90 values for glycopeptide and tigecycline. Although strains isolated from patients in all age groups remained sensitive to vancomycin, strains isolated from patients in the old-old and longevity groups demonstrated resistance to teicoplanin. The MIC90 for tigecycline was the highest in the group aged >90 years.

**Conclusions:**

MRSA constitutes a significant epidemiological problem in cases of hospital-treated pneumonia. The findings were similar for long-term-care facilities, where MRSA appears to affect male residents in particular, although there were fewer male residents than female residents. The low sensitivity to TMP/SXT of SA strains isolated from the oldest patients indicates potentially serious challenges pertaining to efficacious treatment of SA infections.

## Background

The proportion of the population aged ≥65 years is rising steadily. It is estimated that by 2025 the number of such people will increase by more than two-fold [1]. The older population (>65 years) is anticipated to exceed 1 billion persons by 2030 [2]. The average age of European individuals is already the highest in the world. In 2000, people aged ≥65 years represented 14% of the population, which is expected to increase to 25% of the population by 2050 [3]. Thus, challenges associated with infections in older patients and their impact on medical and socioeconomic systems in developing countries require specific assessment [2, 4]. An increased burden of infection in the older is linked to age-related dysfunction of the immune system, malnutrition and anatomic and physiological modifications. Antimicrobial therapy is often less effective in older patients owing to the rare initiation of empiric therapy and late diagnosis because older individuals often exhibit fewer signs and symptoms of infections.

*Staphylococcus aureus* (SA) is a microorganism that comprises normal human flora with the capacity to cause serious infections. SA primarily colonizes warm and moist regions of mucous membranes, especially the nasal vestibule, where it is located. Methicillin-resistant SA (MRSA) is defined by resistance to penicillin derivatives and other antimicrobial agents containing beta-lactam rings and remains one of the principal multidrug-resistant bacterial pathogens causing infections, especially nosocomial infections. MRSA infections are associated with a worse prognosis than non-MRSA infections [5, 6]. Methicillin resistance in SA is usually accompanied by resistance to other groups of antimicrobial agents, thus therapeutic options are sometimes limited to glycopeptides or linezolid [7, 8]. In Poland, the prevalence of MRSA is regional; in 2013, non-teaching hospitals in southern Poland demonstrated a frequency of 15.1% [9]. However, MRSA presents a significant problem for older patients, especially those in institutional settings [10].

The aim of the study was to assess the prevalence of MRSA in older populations in outpatient care facilities, hospitals and long-term care facilities (LTCF) in the provinces of Malopolska and Silesia and clarify the effect of age on the prevalence of MRSA.

## Methods

This retrospective cohort study analyzed a database containing the results of laboratory data from three cooperating laboratories in southern Poland: The Chair of Microbiology, Jagiellonian University Medical College in Krakow; KORLAB NZOZ in Ruda Slaska; and the Microbiological Laboratory, St. Barbara Regional Hospital in Sosnowiec between January 1, 2013 and December 31, 2013. The study included only those records that meet the following criteria: patients aged ≥60 years treated for SA infections between January 1, 2013 and December 31, 2013. Patients with SA infections were defined according to the diagnosis of the physicians, who collected information regarding age, sex, type of infection and place of infection treatment.

Drug resistance was determined using two methods: manually using the Kirby-Bauer disc diffusion method on Mueller-Hinton agar plates and automatically using the Vitek-2® system (bioMérieux, Marcy l’Etoile, France). Results were interpreted according to European Committee on Antimicrobial Susceptibility Testing, EUCAST clinical breakpoints [11]. All SA strains were tested for resistance to commonly used antimicrobial agents (erythromycin, clindamycin, amikacin, gentamicin, tobramycin, moxifloxacin, ciprofloxacin, tetracycline, trimethoprim/sulfamethoxazole (TMP/SXT)). Quantitative assay for determining the minimum inhibitory concentration (MIC) of glycopeptides was performed using the vancomycin/teicoplanin MIC Test Strip (Liofilchem, Argenta, Poland). MIC trends were assessed as MIC50 and MIC90 values (MICs required to inhibit the growth of 50 and 90% of organisms, respectively) [12].

### Statistical analysis

Analyses were performed using StatSoft Statistica software (StatSoft Inc., version 10, Dell Statistica, Tulsa, OK, USA). The odds ratio (OR) and 95% confidence interval (95%CI) were calculated. The normality of the distribution of continuous variables was tested using the Shapiro-Wilk test. No variables had normal distributions; thus, variables were presented as medians and 25th (Q1) and 75th (Q3) percentiles. Continuous variables were tested using the Mann-Whitney *U* test or Kruskal-Wallis test with appropriate post-hoc tests. For dichotomous variables, the Chi-square test was used for expected frequencies >10, the Chi-square test with Yates’ correction was used for expected frequencies between 5 and 10 and the Chi-square test with confirmation by Fisher’s exact test was used for expected frequencies of 5 or lower. Values of *p* < 0.05 were considered statistically significant.

## Results

In total, the study population included 613 participants. Participants were categorized by age as follows: young old: 60–74 years, *N* = 311; late old age (old old): 75–85 years, *N* = 272; and oldest age (longevity): ≥85 years, *N* = 30. The median age of the studied population was 72 years (Q1;Q3: 66;79 years). The median age for male and female patients were 70 and 74 years, respectively (*p* < 0.0001, OR 0.32, 95%CI 0.137–0.769) (Table 1).

**Table 1:** The demographic characteristics of the study group

Characteristics	Total (*N* = 613)	Male (*N* = 289)	Female (*N* = 324)	OR (95%CI)	*P*-value
Age (years) (median, quartiles)	72 (66;79)	70,0 (65;77)	74 (66;80)	N/A	0.0003
Step aging *n* (%)					<0.0001
Young old	311 (100.0)	172 (55.3)	139 (44.7)	1.9 (1.42–2.69)	
Old old	272 (100.0)	110 (40.4)	162 (59.6)	0.4 (0.27–0.52)	
Longevity	30 (100.0)	7 (23.3)	23 (76.7)	0.3 (0.14–0.77)	
Type of infection *n* (%)					0.0231
PNU	47 (100.0)	22 (46.8)	25 (53.2)	0.9 (0.54–1.79)	
BSI	37 (100.0)	25 (67.6)	12 (32.4)	2.3 (1.09–4.60)	
SSTI	416 (100.0)	196 (47.1)	220 (52.9)	0.9 (0.71–1.39)	
EI	62 (100.0)	30 (48.4)	32 (51.6)	1.1 (0.63–1.79)	
Others	51 (100.0)	16 (31.4)	35 (68.6)	0.5 (0.26–0.89)	
Place of the treatment infections *n* (%)					
INPATIENTS	430 (100.0)	213 (49.5)	217 (50.5)	1.4 (0.97–1.96)	0.0309
LTCF	16 (100.0)	3 (18.8)	13 (81.6)	0.3 (0.07–0.89)	
OUTPATIENTS	167 (100.0)	73 (43.7)	94 (56.3)	0.8 (0.58–1.18)	
Infections treated in hospitals (INPATIENTS *N* = 430, *n* (%))					
ICU	19 (100.0)	12 (62.3)	7 (36.8)	1.8 (0.69–4.64)	0.014
non-ICU	411 (100.0)	201 (48.9)	210 (51.1)		

*Abbreviations*: *PNU* pneumonia, *BSI* bloodstream infection, *SSTI* skin and soft tissue infection, *EI* eye infection, *INPATIENTS* Hospital Infection, *LCTF* long term care facility, *OUTPATIENTS* Community Infection, *ICU* Intensive Care Unit, *Non-ICU* Non Intensive Care Unit, *OR* odds ratio, *95%CL* 95% confidence interval, *P-value* probability value, *N/A* not applicable/ not available?

Patients treated in hospitals for SA infection were usually treated in non-intensive care unit (ICU) departments (*n* = 309). Bloodstream infections (BSI) accounted for 6% of all infections and were twice as frequent in males than in females (*p* = 0.0231, OR 2.25, 95%CI 1.098–4.604), despite the predominance of females. Additionally, 12 cases (67%) of BSI and pneumonia occurred in patients aged ≥75 years. Other infections (non-classified, e.g., ear or urinary tract infections) accounted for 8% of all cases, with more than half of the affected patients being female.

The prevalence of MRSA was significantly different in categories of patients: from 14.1% in young old, 19.5% in old old and 26.7 in longevity. MRSA was significantly less frequently reported in cases of pneumonia, 40.4% of SA strains (*p* < 0.0001, OR 0.3, 95%CI 0.14–0.49). The nosocomial MRSA infections were more common in ICU departments: prevalence 36.8%, than in non-ICU departments: prevalence 17.3% (OR 2.8, 95%CI 1.06–7.34, *p* = 0.014). Bloodstream infections, which accounted for 6% of all infections, were more frequent in males (*p* = 0.0231, OR 2.25, 95%CI 1.098–4.604). Additionally, MRSA tended to be associated with BSI while methicillin-susceptible SA (MSSA) was associated with other infection types, although these associations were not significant (Table 2). MRSA infections were significantly less frequent among LTCF residents (*p* = 0.0033, OR 0.3, 95%Cl 0.09–0.69), whereas MSSA infections were significantly more frequent among patients in non-hospital settings (*p* = 0.0033, OR 1.7, 95%CI 1.03–2.92) (Table 2).

**Table 2:** Features of the etiology of infections caused by *Staphylococcus aureus*

Characteristics		Total (*N* = 613)	MSSA (*N* = 508)	MRSA (*N* = 105)	OR (95%CI)	*P*-value
Age (years) (median, quartiles)		72 (66;79)	75 (67;81)	72 (65;78)	N/A	0.0048
Gender:	Female	322 (100.0)	214 (82.3)	57 (17.7)	1.4 (0.93–2.16)	0.5909
	Male	291 (100.0)	255 (83.5)	48 (16.5)		
Step aging *n* (%)						0,0849
Young Old		311 (100.0)	267 (85.9)	44 (14.1)	1.5 (1.00–2.35)	
Old Old		272 (100.0)	219 (80.5)	53 (19.5)	0.7 (0.49–1.13)	
Longevity		30 (100.0)	22 (73.3)	8 (26.7)	0.6 (0.24–1.27)	
Disease *n* (%)						<0.0001
PNU		47 (100.0)	28 (59.6)	19 (40.4)	0.3 (0.14–0.49)	
BSI		37 (100.0)	27 (73.0)	10 (27.0)	0.5 (0.25–1.14)	
SSTI		416 (100.0)	350 (84.1)	66 (15.9)	1.3 (0.85–2.03)	
EI		62 (100.0)	56 (90.3)	6 (9.7)	1.7 (0.72–4.06)	
Others		51 (100.0)	47 (92.2)	4 (7.8)	2.6 (0.91–7.31)	
Place of the treatment infections *n* (%)						0.0033
INPATIENTS		430 (100.0)	352 (81.4)	78 (18.1)	0.8 (0.49–1.26)	
LTCF		16 (100.0)	9 (56.3)	7 (43.8)	0.3 (0.09–0.69)	
OUTPATIENTS		167 (100.0)	147 (88.0)	20 (12.0)	1.7 (1.03–2.92)	
Infections treated in hospitals (INPATIENTS *N* = 430, *n* (%))						
ICU		19 (100.0)	12 (63.2)	7 (36.8)	2.8 (1.06–7.34)	0.014
non-ICU		411 (100.0)	340 (82.7)	71 (17.3)		

*Abbreviations*: *PNU* pneumonia, *BSI* bloodstream infection, *SSTI* skin and soft tissue infection, *EI* eye infection, *INPATIENTS* Hospital infection, *LCTF* long term care facility, *OUTPATIENTS* Community Infection, *ICU* Intensive Care Unit, *Non-ICU* Non Intensive Care Unit, *OR* odds ratio, *95%CL* 95% confidence interval, *P-value* probability value, *MSSA* Methicilin Sensitive *Staphylococcus aureus*, *MRSA* Methiciln Resistant *Staphylococcus aureus*, *N/A* not applicable/ not available?

Resistance to macrolides and lincosamides was similar among all three age groups with a slight increase in the longevity group. Resistance to aminoglycosides and fluoroquinolones clearly increased with age (Table 3). The most dramatic increase in resistance was to trimethoprim/sulfamethoxazole (TMP/SXT); resistance increased from 17% of the young old group to over 80% in old-old and longevity groups (Table 3).

**Table 3:** Antimicrobial resistance most often used in treating infections with *Staphylococcus aureus* etiology

Groups of drugs	Antimicrobial	% resistance in the age groups		
		Young Old [*N* = 311]	Old-Old [*N* = 272]	Longevity [*N* = 30]
Macrolides	Erythromycin (15 μg)	25.2	26.1	30.0
Lincosamides	Clindamycin (2 μg)	22.4	22.1	30.0
Aminoglycosides	Amikacin (30 μg)	25.1	23.8	41.7
	Gentamicin (10 μg)	18.8	22.2	33.3
	Tobramycin (10 μg)	29.4	32.7	50.0
Fluoroquinolones	Moxifloxacin (5 μg)	23.7	34.5	48.1
	Ciprofloxacin (5 μg)	20.8	25.2	52.9
Tetracycline	Tetracycline (30 μg)	23.8	18.2	30.0
Other	Trimethoprim/ Sulfamethoxazole (1.25/23.75 μg)	17.0	83.3	81.2
	Linezolid (10 μg)	0	0	0

Comparison of the prevalence of the MRSA phenotype vs. MRSA + MLSB phenotype (resistance to macrolides, lincosamides and streptogramin B) among all three age groups revealed that the longevity group demonstrated significantly higher accumulated resistance compared with the other age groups (*p* = 0.0371). However, no significant difference in antibiotic resistance was observed according to sex (Table 4).

**Table 4:** MRSA vs MRSA + MLSB phenotype depending on age group and gender

		Age *n* (%)			*P*-value
		Young old [*N* = 311]	Old-old [*N* = 272]	Longevity [*N* = 30]	
Gender	Female	139 (45.1)	162 (52.6)	7 (2.3)	<0.0001
	Male	172 (56.4)	110 (36.1)	23 (7.5)	
MRSA		44 (41.5)	53 (50.5)	8 (7.6)	0.0849
MRSA + MLSB		34 (41.5)	40 (48.8)	8 (9.8)	0.0371

*Abbreviations*: *MRSA* Methicillin-Resistant *Staphylococcus aureus*, *MLSB* Macrolide, Lincosamide and Streptogramin B Resistance

All age groups demonstrated increased MIC90 values for glycopeptide and tigecycline. Although strains isolated from patients in all age groups remained sensitive to vancomycin, strains isolated from patients in the old-old and longevity groups demonstrated resistance to teicoplanin. The MIC90 for tigecycline was the highest in the group aged >90 years (Table 5).

**Table 5:** MIC [mg/ml] values for drugs “last resort” of the MRSA patients in different age categories

„Last-resort” drugs	Young old	Old-old	Longevity
Vancomycin [*N* = 136]			
MIC 50	0.75	0.75	0.5
MIC 90	1	1	1.5
Teicoplanin [*N* = 136]			
MIC 50	1	1	0.75
MIC 90	2	3	3
Tigecycline [*N* = 230]			
MIC 50	0.094	0.094	0.094
MIC 90	0.19	0.25	0.38

*Abbreviations*: *MIC 50* minimum inhibitory concentration, that inhibits 50% of bacterial isolates, *MIC 90* minimum inhibitory concentration, that inhibits 90% of bacterial isolates

## Discussion

The occurrence of MRSA in Europe displays large inter-country variations [ECDC 2013]. A majority of the countries reported frequencies below 20%, which has significantly decreased over the last 4 years [13]. The frequency of MRSA infection is generally lower in northern Europe and higher in southern and south-eastern Europe, however, increasing trends were observed for four countries: Germany, Poland, Portugal and Romania [13]. MRSA and MSSA are also frequently resistant to fluoroquinolones. The report concluded that MRSA remains a public health priority and recommended comprehensive MRSA strategies targeting all healthcare sectors (acute and ambulatory care settings and LTCF) to curb the spread of MRSA in Europe.

It is well known that infections in older patients are a critical medical problem [2]. Older persons generally have greater susceptibility to infections than younger adults because aging is associated with immune dysfunction, especially in cell-mediated immunity [14]. Older patients often require hospitalization and unfortunately contribute substantially to the influx of multidrug-resistant organisms (MDRO) into the hospital setting [15, 16].

Additionally, MRSA presents a major problem for older patients [10]. The emergence of bacterial resistance in older patients may be potentially harmful in both long-stay rehabilitation facilities and acute care settings [17]. Individuals colonized with MRSA are at increased risk of MRSA infection and the poor functional status of older patients is associated with MRSA carrier status [18]. The most serious infection of the SA etiology are BSIs, particularly among older male adults, patients with immunity disturbances or comorbidity and also those that have frequent health care contact [19]. Moreover, patients with SA-BSI have an increased risk of developing associated complications, such as acute complications (shock, adult respiratory distress syndrome, disseminated intravascular coagulation) or infective endocarditis and metastatic supportive complications [20]. Many patient factors contribute to the limited therapeutic options available including concomitant diseases, drug-antibiotic interactions, compromised immunity and metabolic insufficiency.

The microbiological profile and acquisition source of BSI have also been studied in older people, however, the majority of these studies made no distinction between younger and older people. Additionally, no distinction was made within the older population itself, and identifying older people at a cut-off age of 65 years, generally corresponding to retirement age, is probably unsatisfactory, especially with regard to infectious disease [21].

Notably, our results demonstrated increasing resistance to TMP/SXT with age. Some investigators recommend TMP/SXT for MRSA infections because of its low cost and familiarity to health care providers as an older antimicrobial [22]. Unfortunately, in our analysis, SA strains isolated from older patients demonstrated significantly increased TMP/SXT resistance, limiting its usefulness in the treatment of patients aged ≥75 years. Lee et al. showed strains isolated from patients with febrile urinary tract infections demonstrating very high resistance to TMP/SXT, no metter how old the patients were [23]. According to a study by Leistevuo et al., TMP/SXT (or trimethoprim alone) was most frequently prescribed antimicrobial medicine to aged ≥85 years females with urinary tract infections (37%) [17]. According to the most recent publications from the ECDC (European Centre of Disease Control and Prevention) on community antibiotic consumption, TMP/SXT is the lowest consumed antimicrobial group (expressed in DDD (define daily dose) per 1000 inhabitants per day) [24] in European countries. However, this ECDC report revealed the highest usage of TMP/SMX in Poland (ranging from 1.0 DDD in 2008 to 1.5 DDD in 2011 per 1000 inhabitants and per day). Finland was the second highest consumer of TMP/SXT among European countries during this period [24]. Unfortunately, the Finnish report did not present antibiotic consumption according to specific age groups. However, high antibiotic prescriptions among older patients was confirmed in some populations [17, 25]. The key issue is appropriateness of the antimicrobial treatment in relation to the laboratory results, especially in general practice. The study results of Vellinga et al. revealed that treatment of uncomplicated UTI was considered appropriate only for 55% of the patients [26]. Moreover, it was also observed high level of TMP/SXT resistance (30,5%) in *E. coli* isolated in UTI [26].

The conducted analysis demonstrated that MRSA remains a major epidemiological and therapeutic problem not only in hospitalized patients, but also LTCF residents. Thus, infection control as practiced in hospitals and LTCF plays a significant role in health care and possibly other institutional settings [27]. However, SA pneumonia is significantly more frequently managed in hospitals in different departments, not only ICUs, and is significantly more often associated with MRSA. The higher MIC values observed for teicoplanin in the longevity group may also prove to be crucial, especially with reference to patients hospitalized in non-ICU departments. According to earlier research conducted by Lee et al. and Holland & Fowler [28], elevated MIC values for vancomycin (>1.5 mg/L in MRSA and >1.5 mg/L in MSSA) have been confirmed as an independent risk factor for 30-day mortality in patients with SA bacteremia. Moreover, MIC values for teicoplanin could be considered a surrogate marker for pathogen-specific factors responsible for worse outcomes or increased virulence secondary to antibiotic resistance [29].

The present study also demonstrated that MRSA infections in LTCF residents affect males more often than females, The results of our other study also showed greater exposure to the MRSA infections in men than women among diabetic patients with foot ulcers [30]. Those findings may reflect lower hygiene standards in the elderly, especially in male gender. Inadequate hygiene habits can increase the risk of horizontal spread, which is typical of MRSA strains [31].

### Study limitations

There are some limitations associated with this study. First, the demographic information of the study population is limited. For example, previous antimicrobial usage, comorbidity, disability and patient outcome data were not available because of the retrospective nature of the study. Additionally, differences between age groups for hospitalization-specific factors could not be assessed because of this data limitation.

## Conclusions

Methicillin-resistant SA (MRSA) constitutes a significant epidemiological problem in cases of hospital-treated pneumonia. The findings were similar for LTCF, where MRSA appears to affect male residents in particular, although there were fewer male residents than female residents. The low sensitivity to TMP/SXT of SA strains isolated from the oldest patients indicates potentially serious challenges pertaining to efficacious treatment of SA infections.

## Abbreviations

95% CL: 95% Confidence interval
BSI: Bloodstream infection
ICU: Intensive care unit
LCTF: Long term care facility
MRSA/ MSSA: Methicillin-Resistant / Methicilin-Sensitive *Staphylococcus aureus*
OR: Odds ratio
*P*-value: Probability value
SA: *Staphylococcus aureus*
TMP/SXT: Trimethoprim/sulfamethoxazole
